# Low-Cost Label-Free Electrochemical Aptasensor for Dual Detection of Dengue and Zika Using Fluorine-Doped Tin Oxide Modified with APTES and Gold Nanoparticles

**DOI:** 10.1021/acsomega.5c08082

**Published:** 2025-11-07

**Authors:** Bassam Bachour Junior, Marina Ribeiro Batistuti Sawazaki, Ricardo Estéfani França Rocha, Éder José Guidelli, Marcelo Mulato

**Affiliations:** † Department of Physics, Faculty of Philosophy, Sciences and Letters, University of São Paulo, 14040-901 Ribeirão Preto, São Paulo, Brazil; ‡ Department of Morphology and Animal Physiology, Faculty of Agricultural and Veterinary Sciences, São Paulo State University, 14884-900 Jaboticabal, São Paulo, Brazil

## Abstract

The incidence of arbovirus infections, such as dengue and zika, has increased dramatically in recent decades, especially in tropical regions and in technologically limited places. The goal of this study is to create an affordable platform for both dengue and zika detection. Fluorine-doped tin oxide-coated substrates were modified with APTES and gold nanoparticles. The modified surfaces were functionalized with the DNA aptamer and 6-mercapto-1-hexanol. Electrochemical impedance spectroscopy was used to characterize and optimize aptasensors’ performance using nonstructural protein 1 (NS1) proteins as biomarkers. The biosensor exhibited a limit of detection (LoD) of 0.21 ng/mL for dengue in phosphate-buffered saline (PBS), with a sensitivity of 19.26% per decade. In commercial human serum, the platform showed a signal increase of 2% per decade, a LoD of 0.55 ng/mL, and a sensitivity of 11.87% per decade. For zika detection, the biosensor presented 0.68 ng/mL LoD and a sensitivity of 26.34% per decade in PBS. In this case, the presence of a biological matrix from commercial human serum induced complex interaction with NS1, resulting in nonlinear sensor responses. Although this platform was not able to distinguish between zika and dengue infections, it enables the detection of both in a single test, representing a promising approach for clinical diagnostics.

## Graphical abstract

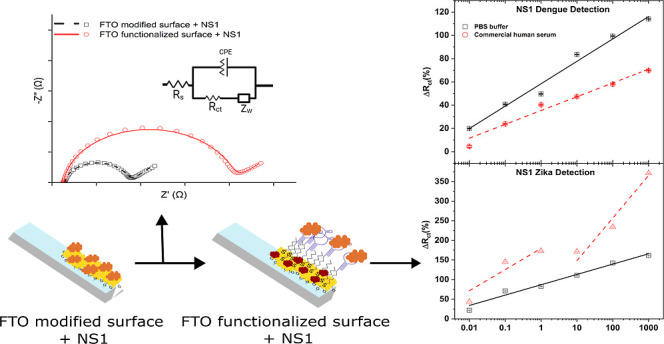

## 1 Introduction

Dengue and zika are tropical diseases that have become a significant public health concern. Both are transmitted by *Aedes aegypti* mosquito, infected with a virus from *Flaviviridae* family. The World Health Organization has classified zika as a major threat to global public health, similar to dengue.^1^ These diseases have common initial symptoms, including fever, rash, and joint pain, which are also consistent with those from influenza. However, dengue can progress to serious conditions, including vascular collapse, shock, and hemorrhages, which require intensive medical monitoring.^2^ Likewise, zika has the potential to cause significant complications not only in adults but also in fetuses from infected pregnant women. These can include microcephaly, macular atrophy, and craniofacial and musculoskeletal malformations.^3,4^ Therefore, the early detection of both diseases is essential to enable appropriate treatment and individualized monitoring.

These virus infections result in the production of different structural and nonstructural proteins. Nonstructural protein 1 (NS1) is particularly interesting due to its extracellular location, which distinguishes it from others. Indeed, it is the only protein secreted into blood plasma, allowing minimal or noninvasive detection.^5^ This protein has been widely employed as a biomarker, particularly in early stages of dengue when antibody production has not reached significant levels.^6^ In these cases, the detection of NS1 corresponds to an important tool for initial diagnosis and can also give information about infection severity, given elevated levels of NS1 in plasma are related to dengue hemorrhagic fever.^7^

Although NS1 is commonly used for dengue diagnosis, some studies have also investigated its potential for zika detection. Traditional methods for zika rely on the detection of antibody or viral RNA through polymerase chain reaction (PCR) due to the different NS1 secretion concentrations in the first or second infection.^8,9^ Gold standard techniques, such as enzyme-linked immunosorbent assay, PCR, and electrochemiluminescence,^7,10,11^ are used and also require specialized laboratories with multistep protocols, lengthy analysis times, expensive instrumentation, and highly qualified personnel.^12^

Parallelly, new technologies have been investigated for low-cost detection. Electrochemical techniques have demonstrated promising results due to their versatility, high sensitivity, and potential for miniaturization. These techniques enable the development of portable and low-cost biosensing platforms, which are particularly suited to difficult access regions. Among these, electrochemical impedance spectroscopy (EIS) has emerged as a particularly effective technique to analyze interface properties and surface interactions with biomarkers.^13–15^

The evolution of molecular biology has led to the emergence of aptamers, a promising bioreceptor alternative to antibodies. Aptamers are small nucleic acid sequences (DNA or RNA) that can be synthesized and selected in vitro using the systematic evolution of ligands by exponential enrichment process. Furthermore, aptamers exhibit high specificity, high affinity, and large thermal and chemical stability.^16^

Fluorine-doped tin oxide (FTO) thin films can be a cost-effective substrate for electrochemical approaches, offering enhanced chemical stability.^17^ FTO has emerged as a low-cost alternative to indium tin oxide (ITO), especially in applications requiring affordable and strong materials.^18^ Lee et al. described the use of FTO as a substitute for ITO, mainly in electrochemical techniques, given that its surface has nanoporous elements that improve its charge conduction capacity. Energetically favorable sites promote the immobilization of recognition biomolecules, improving sensor sensitivity and stability.^19^ Furthermore, the incorporation of gold nanoparticles enables adsorption, and the formation of a more conductive surface allows different functionalizations, including aptamers.^20–22^

As an alternative for a low-cost platform, this study focuses on the development of a biosensor using FTO substrates modified with APTES, gold nanoparticles, and the functionalization with DNA aptamers aiming the detection of NS1 protein from dengue and zika. The results can contribute to the development and innovation of diagnostic platforms for tropical diseases and for use in endemic regions with limited access to high-cost technologies.

## 2 Materials and Methods

### 2.1 Materials

Sodium borohydride (NaBH_4_), acetone (2-propanone), biological pure ethanol, 3-(aminopropyl)­triethoxysilane (APTES), phosphate-buffered saline (PBS) tablet, 6-mercapto-1-hexanol (MCH), bovine serum albumin (BSA), commercial human serum (human male AB plasma—H4522), sodium phosphate dibasic and monobasic (Na_2_HPO_4_ and KH_2_PO_4_, respectively), magnesium dichloride (MgCl_2_), ethylenediaminetetraacetic acid (EDTA), potassium hexacyanoferrate­(II), potassium hexacyanoferrate­(III) and fluorine-doped tin oxide (FTO) thin films deposited on glass were obtained from Sigma-Aldrich. DNA aptamer sequence 5′-HS­(CH_2_)_6_-TTTTAGCGGAT CCGATGGGTGGGGGGGTGGGTAGGATCCGC G-3′ was obtained from Exxtend. Recombinant zika envelope protein (E–Z) (CLZK001) was obtained from Cedarlane, and recombinant zika NS1 protein (NS1-Z) (SPH2015—PKSV 030273) was obtained from Elabscience. Recombinant dengue NS1 protein serotype 4 (NS1—D) was obtained from Abcam (ab181957). Hydrogen peroxide (H_2_O_2_) was provided by Synth. Tetrachloroauric acid (HAuCl_4_·3H_2_O) was obtained from Exodo Cientifica. Poly­(vinyl alcohol) (PVA—(C_2_H_4_O)*x*) and ammonium hydroxide (NH_4_OH) were obtained from VETEC. All aqueous solutions were prepared using 18.2 MΩ·cm ultrapure water (Merck Millipore Direct-Q 5UV) with a Biopak Polisher filter (Merck Millipore, CDUFB-001). All the chemicals were analytical or commercial grade.

### 2.2 Gold Nanoparticles Synthesis

Gold nanoparticles were prepared using a stock solution of PVA (0.05 g/mL), where 250 μL was added to 100 mL of borohydride solution (8.0 mM). After that, this solution was added to 100 mL of tetrachloroauric acid (2 mM HAuCl_4_·3H_2_O) under vigorous stirring and then kept overnight. Finally, the AuNPs colloidal dispersion was dialyzed in Milli-Q water for 5 days with daily water replacement to remove any reactional residues.^23,24^

### 2.3 FTO Modification

FTO substrates were immersed in acetone, pure ethanol, and deionized water in an ultrasonic bath to remove contaminants.^25^ Then, FTO was immersed in hydroxylation solution (H_2_O_2_ + NH_4_OH) diluted in Milli-Q water (1:1:5) and dried in an oven for 30 min at 60 °C.^26^ After that, the electrodes were immersed in APTES solution for 1 h (1%, v/v in pure ethanol), washed with biological pure ethanol, and dried with N_2_ gas. APTES was employed as a molecular connector, given its terminal amine groups provide attachment sites for AuNPs through electrostatic interactions and amine–Au interaction. Compared to other silanes, APTES offers a suitable balance of stability, reproducibility, and compatibility with biosensing architectures.^27–29^ Subsequently, the platform was immersed in AuNP solution for 2 h, washed in Milli-Q water, and dried again using nitrogen gas.

### 2.4 Platform Functionalization

Aptamers were heated for 10 min at 94 °C for thermal denaturation, followed by incubation at room temperature for 40 min to facilitate their renaturation and three-dimensional structure formation, and diluted in immobilization buffer solution (0.8 M PB + 1.0 M NaCl + 5 mM MgCl_2_ + 1 mM EDTA, pH 7). Modified platforms were functionalized coimmobilizing a 100 μL aptamer and MCH solution (10% ethanol) for 16–18 h in a humidity chamber, at 4 °C to form a self-assembled monolayer (SAM).^30^

Three clean solutions (200 mM PB; 10 mM PB + 100 mM EDTA; and 10 mM PB) were dropped on the top of the platform for 5 min each to remove unbounded molecules. To ensure the total thiol coverage, the platform was backfilled with 100 μL of MCH (10% ethanol) for 1 h, followed by 2 h immersion in PBS buffer solution.^31^ Finally, the electrodes were blocked with BSA (0.1%) in PBS or commercial human serum for 30 min. Figure 1 illustrates the protocol including all procedures for FTO modification, functionalization, and final use in detection.

**Figure 1:**
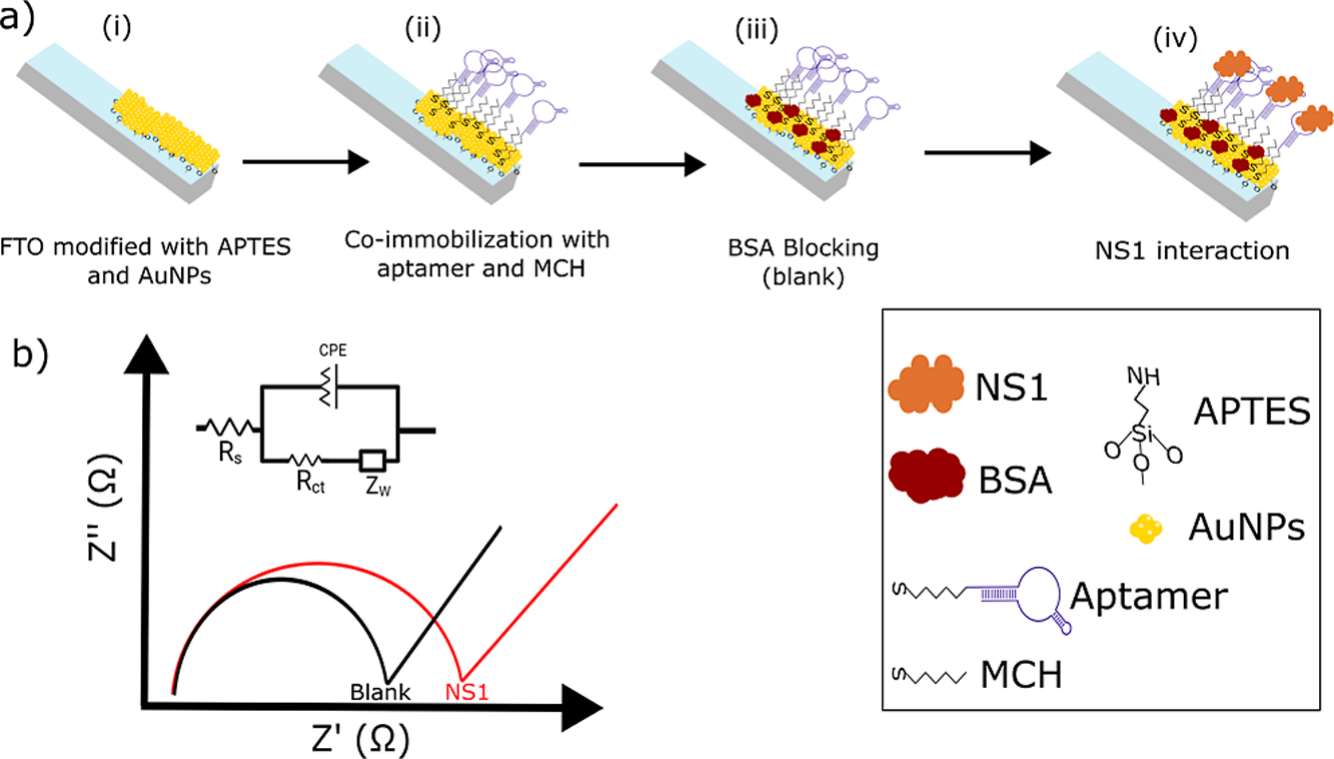
Schematic illustration for the aptasensor protocol. (a) Platform development, including the formation of: (i) FTO modified with APTES and AuNPs, (ii) coimmobilization of the aptamer and MCH on the surface, (iii) BSA blocking, and NS1 interaction with SAM (iv); (b) electrode response obtained from the Nyquist plot of the BSA-blocked surface (blank signal) and after NS1 interaction. Inset: electrochemical equivalent circuit, including solution resistance (*R*_s_), charge transfer resistance (*R*_ct_), constant phase element, and Warburg impedance (*Z*_w_).

### 2.5 Electrochemical Measurements

Electrochemical measurements were performed using a potentiostat (Metrohm/Autolab PGSTAT302) with a three-electrode cell configuration: platinum wire (Metrohm) as the counter electrode, Ag/AgCl (FACTE) as the reference electrode, and modified FTO as the working electrode. All measurements were performed by electrochemical impedance spectroscopy (EIS) using 2.5 mM of hexacyanoferrate II (C_6_FeK_4_N_6_) and 2.5 mM hexacyanoferrate III (C_6_FeK_3_N_6_) in PBS buffer over the frequency range of 100 kHz to 100 mHz with a 10 mV a.c. voltage superimposed on a d.c. bias of open circuit potential. The data were analyzed by fitting the Randle electrochemical equivalent circuit in Nyquist diagram using Autolab software (Nova 2.1).^30,32^

## 3 Discussion and Results

### 3.1 Surface Functionalization and Optimization for Dengue Detection

To develop a functional surface, gold nanoparticles coated with PVA were used due to their ability to promote a more stable colloidal structure, preventing aggregation and enabling a uniform and stable film formation.^24^ However, surface functionalization with thiol-containing molecules—such as aptamers, MCH, or other ligands—may alter the physicochemical and electrical properties of the system. Figure 2 presents the results obtained from target protein from dengue (NS1-D), bovine serum albumin (BSA), and commercial human serum albumin (HSA) interactions with FTO modified with APTES and gold nanoparticles with MCH and without aptamers (Figure 2a) and with MCH and aptamers (Figure 2b). In this case, Δ*R*_ct_ (%) represents the signal variation from the Nyquist diagram. This variation was obtained by measuring the signal from the surface due to the adsorption of a target (*R*_ct(target)_) in comparison with a signal of a blank surface (*R*_ct(blank)_). The signal percentage variation is calculated as Δ*R*_ct_(%) = (*R*_ct(target)_ – *R*_ct(blank)_/*R*_ct(blank)_). All *R*_ct_ data were obtained from fitted equivalent circuit with chi-square test values smaller than 10^–2^.

**Figure 2:**
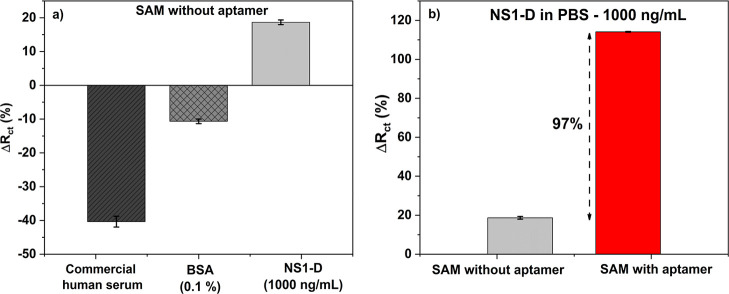
Specifically and nonspecifically platform interactions. (a) Results for different proteins (Human serum, BSA, and NS1-D) interactions with a platform surface without an aptamer, only MCH, or nonfunctionalized SAM; (b) functionalized platform, or SAM with aptamer, and its interaction with NS1-D protein in PBS buffer compared with a nonfunctionalized surface. All experiments were performed in triplicate to obtain error bars.

Gold nanoparticles characterization and interaction with FTO substrates were previously explored and optimized.^24^ Self-assembled monolayers (SAMs) on gold substrates allow a rapid interaction with sulfur-containing molecules, resulting in the formation of stable layers.^33^ All proteins depicted in Figure 2a have sulfur-rich cysteine residues and the potential to interact with gold nanoparticles, resulting in false positive signals.^34–36^ For this reason, all of the proteins were tested individually without an aptamer over the surface. HSA present in commercial human serum exhibited −40.4 ± 1.6%, while BSA displayed a value of −10.6 ± 0.6%. These results are related to the electrostatic influence from ions on the surface of the platform and the amino acid from proteins. In contrast, the NS1-D protein presented a larger signal (18.7 ± 0.7%) compared to the previous ones, indicating an effective interaction of cysteine residues and surface blocking. However, this corresponds to nonspecific interaction.

Khan described the construction of biosensors as requiring a chemically and physically stable self-assembled monolayer (SAM).^37^ The incorporation of gold nanoparticles facilitates surface functionalization via well-established gold–thiol interactions, increasing the surface area.^33,38^ The thiolate molecules possess alkyl chains with SH terminations, thereby enabling covalent interactions with the gold nanoparticles. The present SAM was assembled using DNA chains, or aptamers, modified with thiol groups and coimmobilized with MCH with an optimized ratio of 1:250 (aptamer/total thiol). Figure 2b depicts the formation of the SAM and its interaction with 1000 ng/mL of NS1-D protein. The results demonstrate that the presence of SAM resulted in a notable enhancement of 97% (114.1 ± 0.2%) in the Δ*R*_ct_ signal in comparison to the signal obtained for NS1-D protein on the surface without SAM (Figure 2a). This increase indicates an effective and specific interaction between NS1-D and the DNA aptamers present on the surface.

Pellitero and Arroyo-Currás discussed how the organization of self-assembled monolayers, composed by aptamers and spacer molecules (MCH), contributes to the formation of a more stable surface for analyte target detection.^39^ The presence of phosphate groups in the oligo molecules facilitates electrostatic repulsion when the surface is saturated with aptamers. In contrast, the absence of these chains leads to a loss of the SAM blocking capability, thereby exposing the gold to nonspecific interactions.^40^ Figure 3a presents the results obtained for different aptamers to total thiol ratios (aptamer + MCH) in immobilization buffer (1:100; 1:250; and 1:500) for three different concentrations of NS1-D protein (10, 100, and 1000 ng/mL). Among the evaluated proportions, the 1:250 ratio demonstrated the most favorable performance and linear response (10 ng/mL—14.9 ± 0.7%; 100 ng/mL—34.5 ± 0.6%; and 1000 ng/mL—60.7 ± 0.5%), indicating that the SAM organization is favorable to NS1-D protein interaction.^41,42^

**Figure 3:**
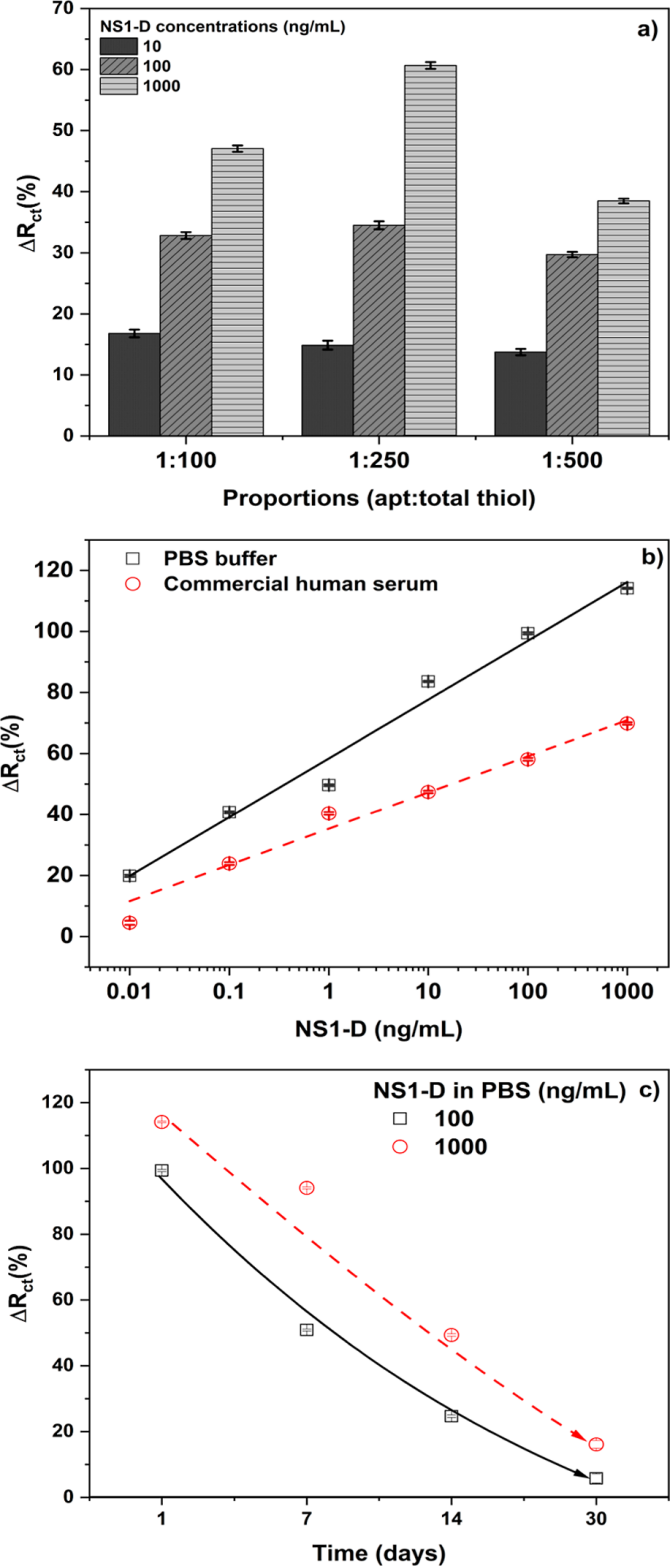
Biosensor optimization and analytical curve for dengue detection. (a) The charge transfer resistance (Δ*R*_ct_ (%)) for each aptamer/total thiol ratio with three NS1-D concentrations (10, 100, 1000 ng/mL) diluted in PBS; (b) analytical curve for NS1-D (0.01–1000 ng/mL) diluted in PBS buffer and commercial human serum; (c) SAM stability over time storage in days. All samples were prepared in triplicate for statistical analysis.

Self-assembled monolayer optimization enables the analytical calibration curve evaluation, a crucial step in biosensor development, and allows the limit of detection (LoD), limit of quantification (LoQ), linear response, and sensitivity. For this purpose, 100 μL of different NS1-D concentrations (0.01 to 1000 ng/mL) was tested. Figure 3b shows the Δ*R*_ct_ (%) for NS1-D concentrations diluted in PBS buffer and commercial human serum. The LoD and LoQ values were determined according to methods previously described in the literature.^43,44^ The LoDs obtained were 0.21 ng/mL with a sensitivity of 19.26 ± 1.42% per decade and 0.55 ng/mL with a sensitivity of 11.87 ± 0.91% per decade, for NS1-D in PBS buffer and commercial human serum, respectively. The LoQs obtained were 0.69 and 1.81 ng/mL, respectively. The linear range of both curves encompasses the complete range (0.01 to 1000 ng/mL), with the equation *Y* = 58.39 + 19.26*X* (*R* ^2^ = 0.978) and *Y* = 35.34 + 11.87*X* (*R* ^2^ = 0.977) for PBS and human serum, where *X* represents the concentration of NS1-D in both equations. The difference verified in Figure 3b indicates the matrix effect over the biosensor, as was also observed in Figure 2.

The long-term storage stability of the biosensor is another critical factor to ensure the reproducibility and reliability of biosensors for extended use. As described by Love and Bossard-Giannesini, thiol-based SAMs have excellent long-term stability, exhibiting only minimal influence from the environmental conditions to which the biosensor is subjected, including humidity and temperature.^45,46^ Figure 3c shows the results for two NS1-D concentrations (100 and 1000 ng/mL) over time, exhibiting 99.4 ± 0.4% and 114.1 ± 0.22% deviation from the initial charge transfer resistance, respectively. Over time, the signal gradually decreased, reaching minimum values of 5.8 ± 1.4% and 16.1 ± 1.2% for the 100 and 1000 ng/mL concentrations, respectively, after 30 days. This degradation may be attributed to the reorganization of molecules on the surface, which can result in a reduction in the efficiency of specific interactions. These findings suggest that the platform provides a sufficiently stable SAM for short- and medium-term applications; however, further studies are required to ascertain its suitability for long-term applications.

Table 1 presents a comparative analysis using all data obtained in this study for dengue detection using NS1 alongside the recent studies from the literature, with a particular emphasis on electrochemical platforms, in the last 3 years. Most of the identified studies employ antibodies as the recognition element, resulting in an enhanced sensitivity. While this is a notable advantage, it is important to consider that antibodies exhibit lower stability than aptamers, particularly under external stress conditions such as temperature and pH variations. Compared with peptides, aptamers offer a broader linear detection range, especially at lower concentrations as well as lower detection limits. It is also important to notice that the biosensors presented in Table 1 were tested with PBS buffer instead of serum, which is closer to real samples and reduces sensitivity, increasing LoD.

**Table 1:** Comparison of the State of the Art for Dengue NS1 Detection Based on the Electrochemical Platform in the Last 3 Years

receptor	linear range (ng/mL)	sensitivity	limit of detection(g/mL)	solution	refs
aptamer	0.01–1000	19.26 ± 1.42% per decade	0.210 n	PBS buffer	this study
aptamer	0.01–1000	11.87 ± 0.91% per decade	0.550 n	commercial human serum	this study
antibody	0.0001–1000	^a^	0.023 p	PBS buffer	^47^
antibody	0.01–1000	^a^	1.630 p	PBS buffer	^48^
antibody	1.29–625	^a^	1.290 n	PBS buffer	^49^
antibody	1–200		22.43 n	commercial human serum	^50^
peptide	0–7500	22.53 Hz mL/μg	0.091 μ	PBS buffer	^51^
nanoparticle	0.0001–0.1	^a^	1.36 p	PBS buffer	^52^

a Data not shown.

### 3.2 Zika NS1 Protein Detection

There is substantial geographic overlap between dengue and zika occurrences around the world, which makes the development of a biosensor for detecting both infections interesting. The same biosensor platform optimized for dengue was tested for zika, and the high sensitivity observed for the NS1 protein was also demonstrated with the zika NS1 protein (NS1-Z). Figure 4a presents the analytical curve, Δ*R*_ct_ (%) as a function of NS1-Z concentrations (0.01 to 1000 ng/mL), diluted in PBS buffer and commercial human serum. The limit of detection (LoD) for zika in PBS was 0.68 ng/mL, with a sensitivity of 26.34 ± 2.00% per decade. The linear range exhibited a linear regression of *Y* = 86.81 + 26.34*X*, with an *R* ^2^ equal to 0.977.

**Figure 4:**
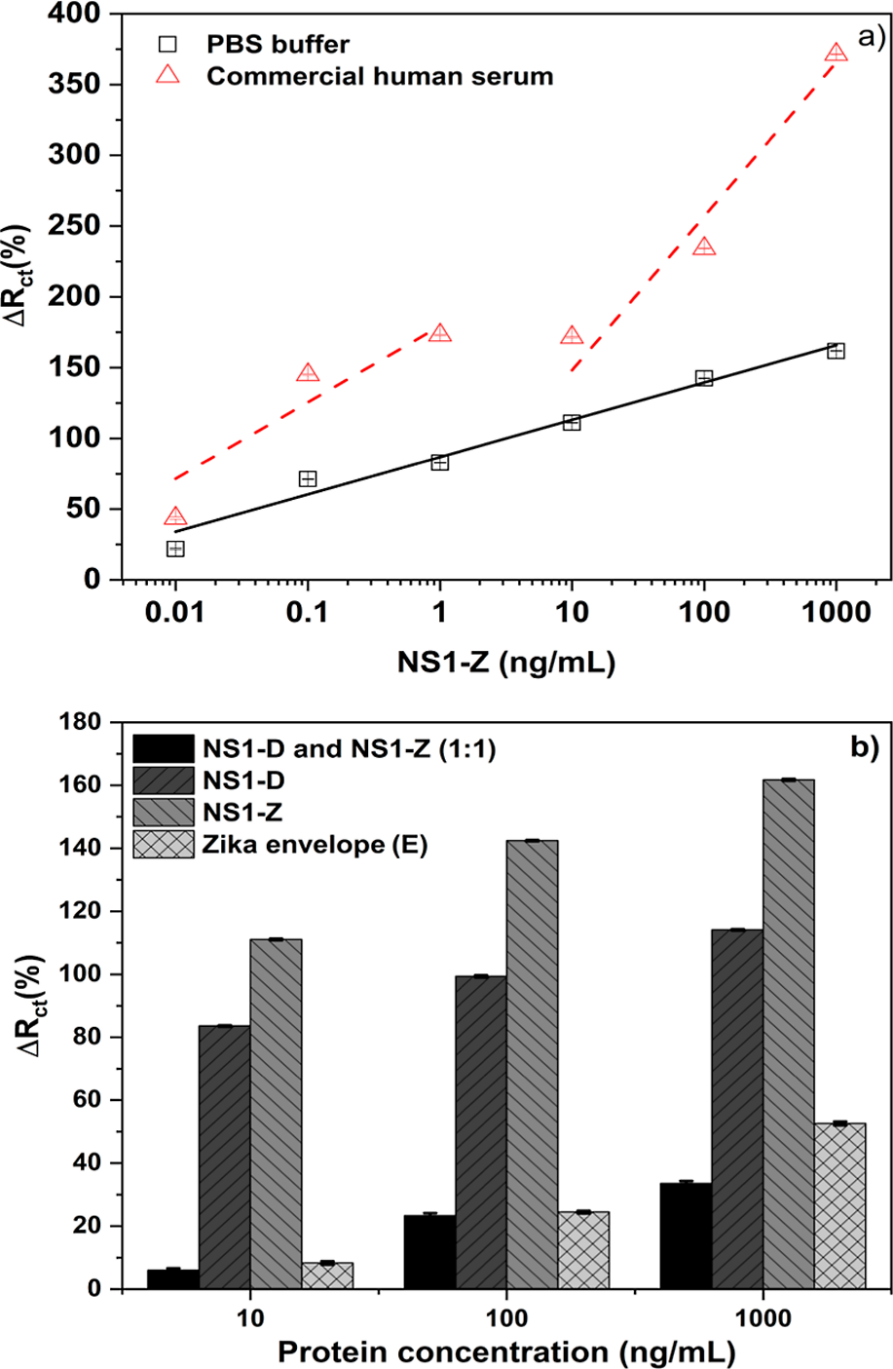
(a) Biosensor analytical curve for zika detection showing the variation in charge transfer resistance (Δ*R*_ct_ (%)) for zika NS1 at different concentrations (0.001 to 1000 ng/mL) in 1:250 (Apt/total thiol) ratio; (b) different concentrations (10, 100, and 1000 ng/mL) of NS1 from dengue (NS1-D), NS1 from zika (NS1-Z), and a mixture of both NS1 proteins (1:1) and zika envelope (E) protein. All samples were prepared in triplicate to construct the error bars.

The results obtained for commercial human serum did not exhibit a monotonic behavior, as evidenced by the low *R* ^2^ values (*R* ^2^ < 0.92). Almeida et al. observed a similar response when analyzing the detection of zika NS1 protein in human serum with another aptamer sequence.^53^ In this case, aptasensor response exhibited dual behavior and/or mechanisms curve. At concentrations below 1 ng/mL, the data suggest a linear and gradual interaction mechanism, with starting point at 43.62 ± 0.83% and a maximum at 173.03 ± 1.4%. This can be related to direct adsorption of NS1 protein on the SAM. In this concentration range, the sensor appears to operate in a regime where the surface is far from being saturated, thereby enabling NS1 to interact with greater specificity with the aptamers.^54,55^ In contrast, at concentrations above 10 ng/mL, an exponential signal increase is observed (171.37 ± 1.45% to 371.45 ± 2.22%), indicating a secondary mechanism.

In complex matrices such as human serum, biomolecules such as lipoproteins can promote secondary interactions between NS1 proteins and the surface of the sensor, potentially leading to cooperative or competitive binding effects.^56,57^ Muthukumaran and Sankararamakrishnan, through simulations, demonstrated that NS1-Z has a greater potential for specific interaction than NS1-D.^58^ These interactions can cause temporary blockage of the protein recognition sites or promote NS1 conformational rearrangements. This could explain the two response mechanisms observed in the aptasensor: at low concentrations, the reduced amount of NS1-Z compared to the complexes present in the serum favors the formation of these associations, limiting direct interaction with the aptamer; at high concentrations, the abundance of NS1-Z prevents the formation of new complexes, in addition to displacing those already present on the sensor surface, freeing up sites for direct interaction and causing an abrupt increase in the signal. In contrast, NS1-D has a lower affinity to interact with these biomolecules present in serum, resulting in a more linear response. Despite the potential diagnostic of zika, Morales et al. note that there are still insufficient clinical data to establish a reliable detection range. However, NS1 production in the early stages of infection represents a promising approach for diagnosing the disease.^59^

The current geographic overlap between the infections also allows coinfection, where individuals can be infected with both viruses concurrently. Zika and dengue are flaviviruses, and there is some degree of cross-reactivity between them.^58^ Figure 4b compares the biosensor response (Δ*R*_ct_) from dengue, zika, and a mixture of both NS1 proteins in the same proportion (1:1) for different concentrations (10, 100, and 1000 ng/mL). A significant difference is observed between dengue and zika signals, with the platform showing, on average, 39% larger signal intensity for zika NS1 compared to dengue NS1. When analyzing the signal with both proteins (83.61 ± 0.23% for dengue and 111.09 ± 0.26% for zika), there is a sharp drop to 5.9 ± 0.6% at a concentration of 10 ng/mL. This behavior is repeated at higher concentrations (1000 ng/mL), where the signals for dengue and zika (114.12 ± 0.22% and 161.71 ± 0.32%, respectively) are significantly larger than the signal for the mixture (33.53 ± 0.79%).

While the platform does not offer selectivity between dengue and zika NS1 proteins, it plays a crucial role in the early diagnosis of both diseases. Patients diagnosed with dengue must receive immediate treatment, with a particular focus on reducing the most severe symptoms, including organ damage, shock, and plasma leakage. On the other hand, patients diagnosed with zika virus infection at an early stage, particularly pregnant women, should be monitored closely for any potential fetal complications. In regions where early diagnosis does not allow for clear differentiation between the infections, it is recommended that patients without additional risk factors be treated for dengue, while pregnant women should be treated and monitored for possible zika virus infection.^3^ The ability to detect NS1 proteins from both viruses, even without complete differentiation, makes this platform a valuable tool for initial clinical management, particularly in endemic areas where the two infections can coexist.

Figure 4b also compares the response from different concentrations (10, 100, and 1000 ng/mL) of zika proteins, including the envelope (E) and NS1 protein. Jang et al. emphasized the importance of detecting the envelope protein (E) from the zika virus as a potential diagnostic tool, given that this protein remains associated with the host cell following infection.^60^ The data demonstrate that the developed sensor exhibits exclusive selectivity to detect NS1 protein in low (10 ng/mL) and high (1000 ng/mL) concentrations, with a minimum signal difference of 74%. These results reinforce the platform’s high selectivity.

Table 2 presents a comparison between results obtained in this study and those reported in the literature, focusing on electrochemical platforms for zika detection through NS1 in the last 5 years. The results demonstrate that, as observed in the context of dengue, the use of antibodies as bioreceptors improves the sensitivity. However, aptamers allow the detection across a broader concentration range compared to antibodies while maintaining a detection limit that is not significantly lower than those reported in the literature.

**Table 2:** Comparison of the State of the Art for NS1 Zika Detection Based on the Electrochemical Platform in the Last 5 Years

receptor	linear range (ng/mL)	sensibility	limit of detection (ng/mL)	solution	refs
aptamer	0.01–1000	26.34 ± 2.00% per decade	0.68	PBS buffer	this work
aptamer	^a^	^a^	^a^	human serum	this work
aptamer	^a^	^a^	^a^	human serum	^53^
antibody	15.62–1000	^a^	0.54	PBS buffer	^61^
antibody	0.1–100	^a^	0.001	undiluted urine	^62^
antibody	^a^	^a^	0.96 μ	PBS buffer	^63^

a Data not shown.

### 3.3 Aptamer Prediction for Dengue and Zika NS1 Interaction

To support simulation studies of biomolecular interactions, such as those between proteins and aptamers, Jumper et al.^64^ developed an artificial intelligence-based platform for predicting protein structures and their interactions, known as AlphaFold. Figure 5 illustrates the results obtained for interactions of the aptamer with NS1 proteins.

**Figure 5:**
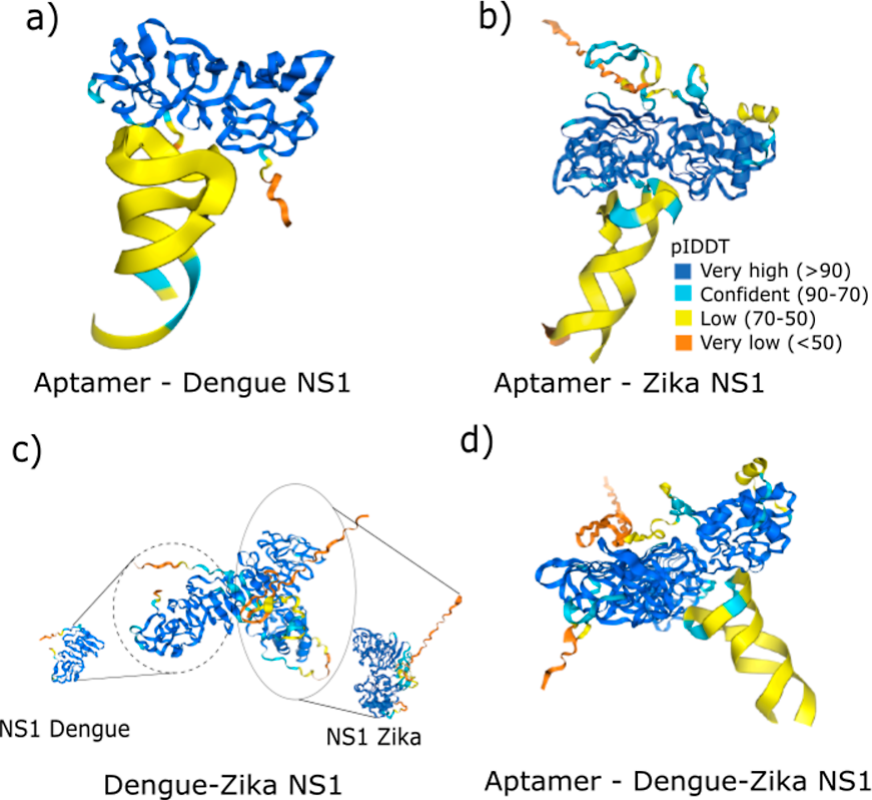
AlphaFold aptamer–protein prediction for interaction between (a) aptamer–NS1-D protein; (b) aptamer–NS1-Z protein; (c) dengue (left) and zika (right); (d) aptamer–dengue and zika NS1.

The analyses obtained by AlphaFold are based on two parameters: the predicted template modeling-score (pTM), which assesses the similarity between two protein structures (ranging from 0 to 1), with values above 0.5 indicating a good prediction of the protein’s native structure; and the interchain predicted template modeling-score quantifies the confidence in the prediction of the interaction between two or more protein chains (ranging from 0 to 1). Higher scores reflect a greater similarity to experimentally observed interactions, with values above 0.5 indicating a reliable interchain interaction prediction.

The interaction between the aptamer and NS1-D demonstrated an ipTM equal to 0.57, indicating a moderate interaction between the two structures. Furthermore, this prediction exhibited a pTM of 0.83, indicating a reasonable reliability from the prediction (Figure 5a). Figure 5b illustrates the aptamer interaction with NS1-Z, exhibiting an ipTM of 0.62 and a pTM of 0.8. These findings suggest a more robust interaction with zika NS1 than with dengue, which is consistent with the data presented and discussed in Figure 4b above.

The analysis of both proteins in Figure 5c indicates an ipTM of 0.85, which corresponds to a strong interaction between them, as well as a pTM of 0.83. The DNA aptamer interaction with this complex dengue/zika is presented in Figure 5d, demonstrating a high degree of confidence in the prediction of the structure (pTM = 0.84) and interaction (ipTM = 0.85). However, this result may suggest that the values are predominantly influenced by interactions between proteins and not including aptamer, given that the signals in the protein mixture were relatively weak. Additionally, this protein interaction occurs between the same aptamer recognition sites, which may prevent the binding sites from being recognized and subsequently reduce the platform’s detection efficiency, as illustrated in Figure 4b.

The findings of this study underscore the significance of detecting NS1 proteins from both dengue and zika viruses, demonstrating the efficacy of the developed biosensor platform and shedding light on the complex biomolecular interactions involved. By enabling rapid and accurate identification of both infections, the proposed approach represents a meaningful advancement in diagnostic strategies—crucial for timely clinical decision-making. Furthermore, the adoption of similar biosensing technologies holds great potential to strengthen epidemiological surveillance and enhance disease control efforts, ultimately contributing to public health improvements in regions affected by these arboviruses.

Despite its encouraging performance, it is imperative to acknowledge the study’s limitations. First, although the biosensor is capable of detecting NS1 proteins from both dengue and zika viruses, it is not yet fully discriminatory when it comes to identifying the presence of these proteins in mixed samples. In order to enhance these results, machine learning was employed to overcome the limitation, with a successful separation of both samples.^65^ Furthermore, the study performed validation using recombinant proteins (NS1) in buffer and serum; therefore, the use of real samples for clinical validation remains an essential next step in the platform’s development. Finally, SAM-based functionalization demonstrated stability for up to 30 days, which may limit long-term deployment. In order to enhance these results, future studies will be conducted using polymers for the protection of SAMs or lyophilization strategies with the objective of extending shelf life and increasing robustness.

## 4 Conclusion

This study focused on the development of a biosensing platform for the detection of dengue and zika viruses through the identification of NS1 proteins along with the investigation of their interactions with a self-assembled monolayer (SAM). The construction of the SAM, followed by electrochemical characterization and optimization using aptamers, enabled the development of a biosensor capable of detecting target analytes at low concentrations, particularly in commercial human serum. However, the presence of serum macromolecules interfered with the recognition of zika NS1, resulting in varied sensor responses that limited the accuracy of the calibration and sensitivity evaluation. Regarding selectivity, the platform did not demonstrate the ability to distinguish between dengue and zika NS1 proteins. Nonetheless, its capacity to detect both infections in a single test represents a significant advancement in clinical diagnostics. This capability can facilitate faster diagnosis and medical decision-making, especially in high-risk groups, such as pregnant women, who are more susceptible to severe complications. Therefore, the present work introduces a versatile diagnostic platform for the detection of multiple tropical diseases with the potential for expansion to other pathogens and applications in low-resource settings with high disease incidence. These findings highlight the strong potential for innovation in the development of miniaturized devices for rapid public health interventions.
